# Cost-Effectiveness of a Community Pharmacist Intervention in Patients with Depression: A Randomized Controlled Trial (PRODEFAR Study)

**DOI:** 10.1371/journal.pone.0070588

**Published:** 2013-08-12

**Authors:** Maria Rubio-Valera, Judith Bosmans, Ana Fernández, Maite Peñarrubia-María, Marian March, Pere Travé, Juan A. Bellón, Antoni Serrano-Blanco

**Affiliations:** 1 Research and Development Unit, Fundació Sant Joan de Déu, Sant Boi de Llobregat, Barcelona, Spain; 2 Mental Health Group, Red de Investigación en Actividades Preventivas y Promoción de la Salud en Atención Primaria, Barcelona, Spain; 3 Department of Health Sciences and Institute for Health and Care Research, Instituut Extra Muraal Geneeskundig Onderzoek, Faculty of Earth and Life Sciences, Vrije Universiteit Amsterdam, Amsterdam, The Netherlands; 4 Clinical and Health Psychology Department, Universitat Autònoma de Barcelona, Barcelona, Spain; 5 Primary Care Health Centre Bartomeu Fabrés Anglada, Servicio de Atención Primaria Delta Llobregat, Àmbit Costa de Ponent, Institut Català de la Salut, Gavà, Spain; 6 Estades en Pràctiques Tutelades Unit, School of Pharmacy, Universitat de Barcelona, Barcelona, Spain; 7 Primary Care Health Centre El Palo, Research Unit of the Distrito de Atención Primaria de Málaga, Departament of Preventive Medicine and Public Health, Universidad de Málaga, Málaga, Spain; Groningen Research Institute of Pharmacy, United States of America

## Abstract

**Background:**

Non-adherence to antidepressants generates higher costs for the treatment of depression. Little is known about the cost-effectiveness of pharmacist's interventions aimed at improving adherence to antidepressants. The study aimed to evaluate the cost-effectiveness of a community pharmacist intervention in comparison with usual care in depressed patients initiating treatment with antidepressants in primary care.

**Methods:**

Patients were recruited by general practitioners and randomized to community pharmacist intervention (87) that received an educational intervention and usual care (92). Adherence to antidepressants, clinical symptoms, Quality-Adjusted Life-Years (QALYs), use of healthcare services and productivity losses were measured at baseline, 3 and 6 months.

**Results:**

There were no significant differences between groups in costs or effects. From a societal perspective, the incremental cost-effectiveness ratio (ICER) for the community pharmacist intervention compared with usual care was €1,866 for extra adherent patient and €9,872 per extra QALY. In terms of remission of depressive symptoms, the usual care dominated the community pharmacist intervention. If willingness to pay (WTP) is €30,000 per extra adherent patient, remission of symptoms or QALYs, the probability of the community pharmacist intervention being cost-effective was 0.71, 0.46 and 0.75, respectively (societal perspective). From a healthcare perspective, the probability of the community pharmacist intervention being cost-effective in terms of adherence, QALYs and remission was of 0.71, 0.76 and 0.46, respectively, if WTP is €30,000.

**Conclusion:**

A brief community pharmacist intervention addressed to depressed patients initiating antidepressant treatment showed a probability of being cost-effective of 0.71 and 0.75 in terms of improvement of adherence and QALYs, respectively, when compared to usual care. Regular implementation of the community pharmacist intervention is not recommended.

**Trial Registration:**

ClinicalTrials.gov NCT00794196

## Introduction

Major depression is a highly prevalent disorder that generates a heavy burden both for the society and the public health system [1]–[3]. Major depression also imposes a substantial financial burden on society through increased health care utilization and absenteeism from paid work [4].

The cost associated with mental disorders currently accounts for approximately 3%–4% of gross domestic product in Europe [5]. In Catalonia, a Spanish region with a population of around 7.5 million inhabitants, the annual cost of major depression in 2006 was 735 million Euros [6]. Productivity loss (indirect costs), accounted for almost the 79% of total costs.

Almost 70% of patients with a mood disorder are prescribed psychotropic drugs, mainly antidepressant [7], [8]. Non-adherence to antidepressants is high, as shown by recently published studies that reported rates of non-adherence of over 75% after 6 months [9]–[11]. This low rates of adherence to antidepressants prevent patients to benefit fully from the effects of the treatment, increasing the costs and the risk of relapse and recurrence [12]–[14].

A number of interventions aimed at improving patients' adherence to antidepressants have been evaluated [15]. Some efforts have concentrated on pharmacists, in their role of drug dispensers and specialists on medication showing a positive effect on adherence to antidepressants [16]. The PRODEFAR study was focused on a community pharmacist's intervention in depressed patients [17]. The pharmacist intervention being evaluated showed to impact positively on patients' health-related quality of life and the impact on the levels of adherence to antidepressants was almost statistically significant in the per protocol analysis. The severity of depressive symptoms was not affected by the intervention [17].

Economic evaluations provide decision-makers with information on how to allocate the limited resources available for health care. However, only one study evaluated the cost-effectiveness of a pharmacist's intervention to improve adherence to antidepressants [18]. The study considered a small sample size both in the main analysis (N = 88) and in the per protocol analysis (only 26 patients in the intervention group). Besides, this study did not include quality adjusted life years (QALY) as a measure of effectiveness thus limiting comparison with other therapies and therapeutic areas [19].

The aim of the present study was to evaluate the cost-effectiveness of a community pharmacist intervention (CPI) in comparison with usual care (UC) for depressed patient initiating treatment with antidepressants in primary care. The economic evaluation was completed from a societal and a healthcare perspective.

## Materials and Methods

Economic evaluation conducted alongside a naturalistic randomized controlled trial with 6 months of follow-up comparing a CPI with UC for patients prescribed a new antidepressant treatment by a general practitioner (GP). A detailed description of the study protocol has been provided elsewhere [17], [20]. The protocol for this trial and supporting CONSORT checklist are available as supporting information; see Checklist S1 and Protocol S1. The study protocol was approved by the Fundació Ethics Committee. Patients signed an informed consent to participate.

### Study population

Participants were recruited in 4 Primary Care Health Centres (30 GPs) from two cities (Gavà and El Prat) in the metropolitan area of Barcelona (1 October 2008–31 May 2011). At first, only the PCHC from Gavà participated in the study but to accelerate patient inclusion, a population from El Prat was included in March 2010. Eligible patients were adults aged 18–75 initiating treatment with antidepressants because of depression. Depression was assessed using the research version of the Structured Clinical Interview for DSM-IV (SCID-I) [21], [22]. GPs were blind to the DSM-IV diagnosis and patient inclusion was performed according to their usual practice.

### Randomization

Randomization was done at the patient level using a computerized random-number generator following a permuted block design. Every GP received a set of 10 sequentially numbered, opaque, sealed envelopes containing patient assignment. The GP sequentially stapled one of the envelopes to the patient's prescription that was opened by the pharmacist in the community pharmacy. Pharmacists were asked to be careful not to use intervention elements in their contacts with the UC group. Patients were asked to avoid conversations concerning the study with other participating patients.

### Interventions

Patients received the CPI when they went to the pharmacy to pick up their first prescription of antidepressants (mean time invested of 14.4 minutes). A shorter version of the intervention was used as a reminder when patients refilled their prescriptions (mean time invested of 7.7 minutes). The CPI consisted of an educational intervention provided by the pharmacist and focused on improving patients' knowledge of antidepressant medication, as well as making patients aware of the importance of compliance to the medication, to reassure the patient about possible side-effects, and to stress the importance of carrying out GPs' advice. Also, in patients with a sceptical attitude towards antidepressants, the intervention aimed to reduce stigma. Pharmacists participating in the study were trained for the intervention.

Patients in the UC group received usual care from their GP and pharmacist. The intervention in the usual care group consisted of filling the prescriptions, addressing patients' questions about medication and giving basic advice about how to take the antidepressant. Pharmacists invested a mean of 7.8 minutes per patient for the first visit and 7.7 minutes for subsequent visits.

### Clinical outcomes

Adherence was measured using electronic pharmacy records. Every time the patient refills his/her medication the system automatically registers the information in the patient's clinical history. This method for assessing adherence provides a good estimate of adherence and has been recommended both in research and clinical contexts [23].

Originally, the intention was for pharmacists to manually register the information on medication dispensed. However, the electronic system was much more reliable and easy to execute and was not affected by the mobility of the patients or the loss to follow-up. The medication possession ratio (MPR) was calculated as (Number of doses refilled/Number of doses prescribed)*100. Some patients abandoned the treatment right after commencement and, after some months without treatment, suffered a relapse and initiated a new treatment. In some cases, the amount of antidepressant drugs refilled by those patients, if we took into account the whole 6-months, period was over the 80%. Consequently, continuity in the acquisition of medication was also checked. Patients were considered to have a drug gap if there was a period of 2 or more months without medication. Poor adherence was defined as having a MPR<80% or having a medication gap [24].

QALYs and severity of depression were assessed at baseline, and 3 and 6 months of follow-up.

Health-related quality of life was measured using the EuroQol-5D [25]–[27] and Spanish tariffs were used to estimate the utility of health states described by the patients [28]. QALYs were calculated by multiplying the utility with the amount of time a patient spent in a particular health state. Linear interpolation was used for transitions between health states.

Severity of depression was assessed using the Patient Health Questionnaire 9-item depression module (PHQ-9) [29], [30]. Remission of symptoms was considered as having a reduction in PHQ-9 scores superior to 50% [31].

### Service utilization and cost measures

Cost data were collected from a societal perspective at baseline, 3 and 6 months. Use of health care resources and lost productivity were assessed with the Client Service Receipt Inventory with a three months recall period [32]. Information about use of psychotropic drugs was collected from computerized pharmacy records. Intervention costs were estimated using the patient study chart kept by the pharmacist. Pharmacists registered the time spend with the CPI and UC patients in each visit to the pharmacy.

A secondary analysis was done from a health system perspective (indirect costs excluded).

Direct healthcare costs comprised visits to publicly and privately funded primary and secondary care providers, hospitalisation, tests, and drugs. The Official Bulletin of the Catalan Government for 2009 was used to estimate the costs of publicly funded services [33]. For privately funded services, we used the information provided by the Official College of Physicians of Barcelona [34].

Indirect costs consisted of the costs of absenteeism from paid work. Costs of work loss were calculated by multiplying the days on sick leave with the minimum daily wage in Spain according to the human capital approach [35].

The unit cost of the community pharmacists was calculated taking into account the pharmacists annum working time as well as general community pharmacy expenses, pharmacists salaries and salaries on costs, taxes and pharmacists annum working time. This information was extracted from a published annual report based on income tax return declarations from Spanish community pharmacies in 2009 [36].

In the CPI group extra costs were included to account for the time spent on the training by the pharmacists. Training costs were estimated by adding the tariffs of the Official College of Pharmacists from Barcelona for similar training courses with the time spent by the pharmacists on the training and taking into account the 12-month incidence of depression in primary care Catalan population.

Table 1 shows the unit costs healthcare resources. Time horizon was less than a year so costs were not discounted.

**Table 1:** Unit costs for healthcare resources in Euros (year 2009 values).

Type of utilisation		Unit costs
Costs in the public health care system	General practitioner	36.0
	Nurse	14.0
	Psychologist	51.6
	Psychiatrist	51.6
	Other medical specialists	51.6
	Hospital emergency visits	142.7
	Hospital stay (per day)	277.6
	Diagnostic tests	Range 3.7–329.0
	Pharmacological treatment	Depending on type and dose
	Social worker	36.0
Costs in the private health care system	Psychiatrist	25.3
	Psychologist	25.3
	Medical specialist	25.3
	General practitioner	25.3
Productivity losses	Abstenteeism from work (Number or net days)	24.0
Intervention costs	Pharmacist (per hour)	68.3
	Extra per-patient cost in the community pharmacist intervention group^a^	5

a In the intervention group an extra 5 € per patient were included to account for the time needed for the training of the pharmacists.

### Statistical methods

The main analyses were done according to the intention to treat principle (ITT). Sample size calculation was based on the primary outcome of the study, i.e. adherence to antidepressants. To observe an improvement of 17 points in the percentage of medication intake and assuming a one-sided alpha of 0.05 and a power of 0.8 a total of 162 patients were necessary. We explored baseline differences between groups with Students t-test, χ^2^-test (or Fisher exact test), and the non-parametric K-sample test on the equality-of-median.

#### Missing data

Fifty-five percent of individuals had at least one missing clinical or cost variable. We cannot be certain about the reasons for the missing data, but no major discrepancy was found between imputed data and complete-case analysis so we are leaning towards its classification as missing at random. Missing values were imputed using multiple imputation by chained equations using the predictive mean matching method. The imputation model included important sociodemographic and prognostic variables associated with the outcome variables and drop-outs (education and presence of depression according to DSM-IV criteria). Fifty imputed databases were created [37].

#### Cost-effectiveness calculations

The incremental costs and effects between groups were modelled by generalized linear models (GLMs) that were fitted with different distribution families (gaussian, inverse gaussian, poisson and gamma) and link functions (identity and log). Akaike and Bayesian information criterion (AIC/BIC) were used to test the models.

For the costs and the QALYs, the gamma and gaussian distribution, respectively, with identity link were the best fit. For adherence to antidepressants and remission of symptoms, a binomial distribution with logit link was used. Sociodemographic and baseline clinical variables considered to be relevant were tested in the models using likelihood ratio tests (p≤0.10). Unadjusted and adjusted analyses are presented.

The overall difference in mean costs and effects between treatments was calculated using Rubin's rules [38]. We calculated the incremental cost-effectiveness ratio (ICER) by dividing the difference in costs between the treatments by the difference in effects.

### Generation of cost-effectiveness planes and cost-effectiveness acceptability curves

To estimate the uncertainty surrounding the cost differences and the ICER, we used bootstrapping with 500 replications in each imputed dataset. Due to the biased and skewed distribution of the costs, a bias-corrected and accelerated (BCa) confidence interval [39] was estimated on each imputed dataset and then averaged.

Bootstrapped cost effect pairs were then plotted on cost effectiveness planes [19] and used to estimate cost effectiveness acceptability curves (CEACs) [40]. Analyses were performed with STATA 12.0.

### Sensitivity analyses

Five sensitivity analyses were conducted to assess the robustness of the results. Firstly, we did a Per Protocol (PP) analysis in which patients in who did not attend to the pharmacy or receive the intervention were excluded. Secondly, we did a complete case analysis without the 52 patients who were lost to follow-up at 6 months. Thirdly, we conducted an analysis where the intervention costs were doubled. Fourthly, we carried out an analysis using the mean salary (52.3€ per day) instead of the minimum salary in Spain. Finally, we did an analysis considering only those patients that fulfilled DSM-IV criteria for depression.

## Results

**Figure 1:**
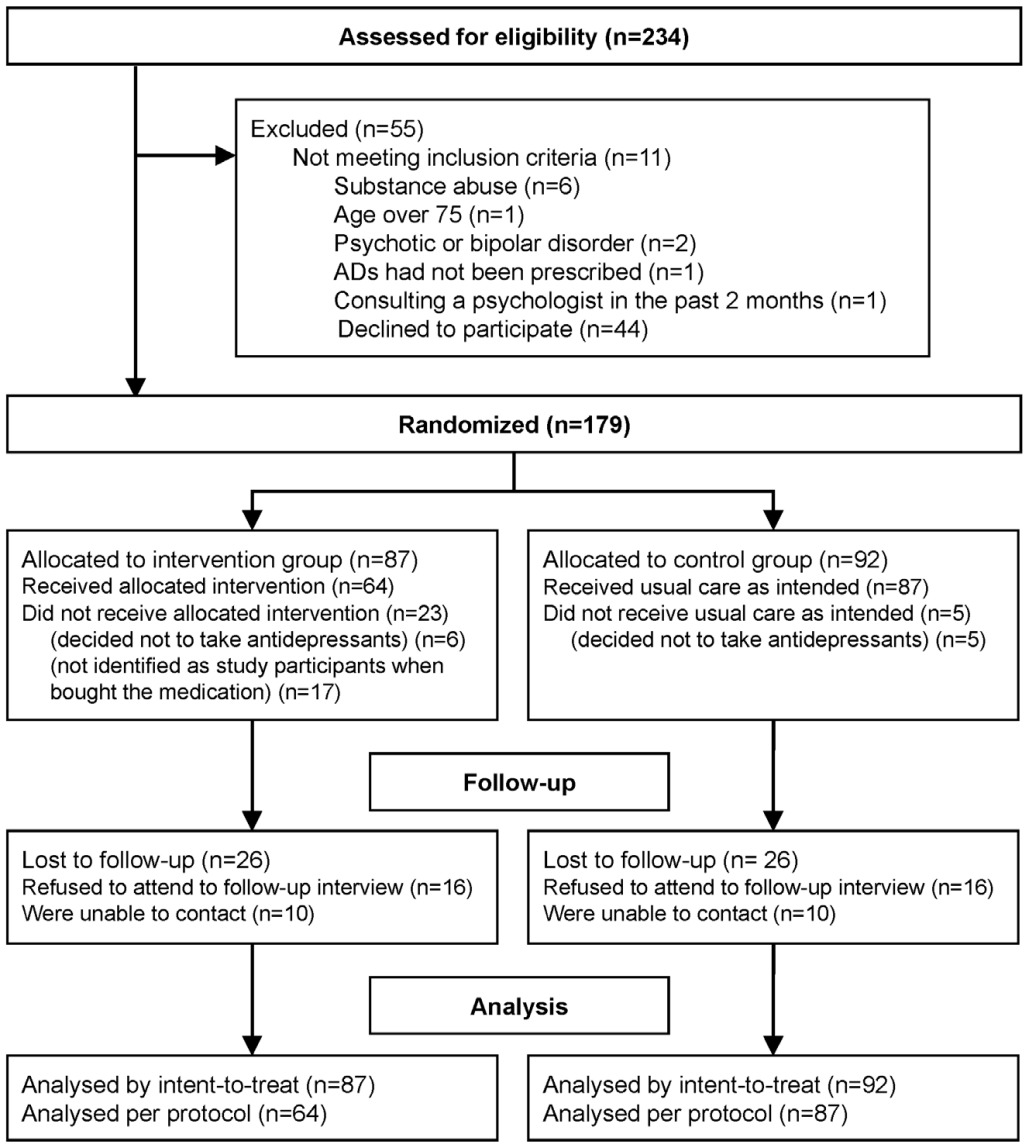
Flow-chart of the study.

Figure 1 shows the flow chart of the study. GPs referred 179 patients that met the inclusion criteria, consented to participate and were randomized to the CPI (n = 87) or UC group (n = 92).

Table 2 shows the baseline characteristics in intervention and control group. Most participants were women (75%), with mean age of 46.6 years. 51% of the participants met DSM-IV criteria for major depression. Statistically significant differences existed in the proportion of women between the two groups. No other baseline differences existed between groups. Sixty-four and 87 patients in the CPI and UC group, respectively, received the intervention as allocated and were included in the PP analysis. 71% of patients attended the 6 months follow-up assessment and were included in the complete-case analysis.

**Table 2:** Socio-demographic and clinical baseline characteristics of the sample.

		Usual care (n = 92)	Pharmacist's intervention (n = 87)
Gender; % women (n)*		83.7% (77)	66.7% (58)
Age; mean (95% CI)		46.3 (43.3–49.2)	46.9 (44.0–48.6)
Marital status; % (n)	Never married	14.1% (13)	18.4% (16)
	Married or living with someone	64.1% (59)	59.8% (52)
	Previously married	10.9% (10)	10.3% (9)
	Widow	10.9% (10)	11.5% (10)
Education; % (n)	No studies	7.6% (7)	5.8% (5)
	Primary	22.8% (21)	23.0%(20)
	Graduated	23.9% (22)	19.5% (17)
	Secondary	26.1% (24)	31.0% (27)
	University	19.6% (18)	19.0% (34)
	Others	–	2.3% (2)
Working status; % (n)	Househusband/housewife	13.0% (12)	17.2% (15)
	Paid employment	40.2% (37)	29.9% (26)
	Paid employment but on sick leave	21.7% (20)	24.1% (21)
	Unemployed	17.4% (16)	16.1% (14)
	Retired	7.6% (7)	9.2% (8)
	Others	–	2.3% (2)
	NS/NC (Missing)		1.2% (1)
Major depression according to DSM-IV criteria; % (n)		50.0% (45)	52.3% (45)
Clinical severity according to PHQ-9; mean (95% CI)^a^		15.8 (14.6–16.9)	16.1 (14.7–17.4)
Number of co-morbidities; % of cases over the median (n)		37.0% (34)	40.2% (35)

* p<0.05.

a PHQ-9 scores can range from 0 to 27, with scores of 15 to 19 corresponding to moderately severe symptoms.

### Cost-effectiveness analyses

Table 3 lists unadjusted costs in the control and intervention groups during 6 months.

**Table 3:** Multiple imputed and pooled costs after 6 months follow-up in the usual care and interventioun groups and mean differences between groups (95% CI) (unadjusted analysis).

Type of cost		Usual care	Intervention	Mean differences
**Direct costs**		409 (303, 515)	412 (322, 502)	3 (−134, 140)
	Visits to primary and secondary care	185 (143, 228)	225 (165, 284)	39 (−27, 106)
	Emergency visits and hospitalisation	113 (49, 176)	86 (39, 134)	−26 (−107, 54)
	Diagnostic tests	61 (30, 92)	44 (26, 63)	−17 (−51, 16)
	Medication costs	50 (38, 62)	57 (44, 69)	7 (−10, 24)
	Intervention costs	16 (13, 20)	32 (27, 37)	16 (9, 22)
**Indirect costs (sick leave)**		342 (110, 573)	647 (351, 943)	306 (−95, 706)
**Total costs**		767 (499, 1035)	1091 (764, 1418)	324 (−97, 745)

Overall costs tended to be higher in the CPI group than in UC patients although not statistically significantly so. The largest part of the cost difference (over 90%) was due to the difference in indirect costs (productivity loss). The intervention costs were statistically significantly higher in the CPI group than in the UC group (Table 3).

Table 4 shows the cost-effectiveness analysis after 6 months follow-up. No statistically significant differences were observed between groups in costs or clinical outcomes, neither in the adjusted or unadjusted analysis (Table 4), although costs were slightly higher in the CPI group.

**Table 4:** Mean pooled differences in total effects and costs at 6 months follow-up and results of cost-effectiveness and cost-utility analyses after 6 months follow-up for the main analysis and for the sensitivity analyses.

	Sample size		Outcome	Cost difference € (95% CI BCa)	Effect difference (95% CI)	ICER/ICUR	Distribution CE-plane			
	I	C					%NE	%SE	%SW	%NW
Main analysis*	87	92	Adherence	74 (−163, 13510)	0.04 (−0.2, 0.1)	1866	63.9	6.9	3.0	26.2
			PHQ-9	74 (−163, 13510)	−0.01 (−0.2, 0.1)	−7651	41.4	4.9	5.0	48.8
			QALY	74 (−163, 13510)	0.01 (−0.02, 0.03)	9872	68.1	7.8	2.1	22.0
Main analysis (unadjusted)	87	92	Adherence	312 (−36, 677)	0.06 (−0.1, 0.2)	5409	73.2	3.9	1.3	21.6
			PHQ-9	312 (−36, 677)	−0.02 (−0.2, 0.1)	−18930	42.2	2.2	3.0	52.6
			QALY	312 (−36, 677)	−0.005 (−0.04, 0.03)	−64181	35.1	2.2	3.0	59.7
Healthcare perspective*	87	92	Adherence	38 (−58, 159)	0.04 (−0.2, 0.1)	962	50.6	20.2	8.9	20.4
			PHQ-9	38 (−58, 159)	−0.01 (−0.2, 0.1)	−3946	32.4	13.9	15.2	38.6
			QALY	38 (−58, 159)	0.01 (−0.02, 0.03)	5092	53.6	22.3	6.8	17.4
Sensitivity analyses*										
Per Protocol analysis	64	87	Adherence	163 (−126, 92275008)	0.11 (−0.2, 0.2)	1455	86.1	3.4	0.5	10.0
			PHQ-9	163 (−126, 92275008)	−0.04 (−0.2, 0.2)	−4350	40.2	2.0	1.9	55.9
			QALY	163 (−126, 92275008)	0.01 (−0.02, 0.04)	25522	60.7	2.8	1.1	35.4
Complete cases	62	65	Adherence	11 (−258, 387)	0.02 (−23.3, 0.2)	696	56.9	7.4	5.2	30.6
			PHQ-9	11 (−258, 387)	0.03 (−0.1, 0.2)	715	56.6	8.6	4.0	30.8
			QALY	11 (−258, 387)	0.02 (−0.01, 0.05)	601	81.4	11.5	1.0	6.0
Double intervention costs	87	92	Adherence	93 (−146, 683)	0.04 (−0.2, 0.1)	2333	65.7	5.1	2.2	27.0
			PHQ-9	93 (−146, 683)	−0.01 (−0.2, 0.1)	−9569	43.0	3.2	4.0	49.7
			QALY	93 (−146, 683)	0.01 (−0.02, 0.03)	12347	70.7	5.1	2.1	22.0
Average salary for absenteeism	87	92	Adherence	159 (−299, 1.1*10^9^)	0.04 (−0.2, 0.1)	3997	66.9	5.6	2.3	25.1
			PHQ-9	159 (−299, 1.1*10^9^)	−0.01 (−0.2, 0.2)	−16392	44.3	3.5	4.4	47.7
			QALY	159 (−299, 1.1*10^9^)	0.01 (−0.02, 0.03)	21152	70.4	5.7	2.3	21.6
DSM-IV criteria for depression	45	45	Adherence	14 (−1.4*10^7^, 6.0*10^9^)	0.02 (−0.1, 0.2)	574	63.4	6.8	3.2	26.6
			PHQ-9	14 (−1.4*10^7^, 6.0*10^9^)	0.03 (−0.1, 0.2)	469	42.9	5.3	4.7	47.1
			QALY	14 (−1.4*10^7^, 6.0*10^9^)	0.0004 (−0.03, 0.03)	32895	67.1			

* Models with costs as dependent variable adjusted for gender, costs in the previous three months and baseline severity of depression. Models with adherence as dependent variable adjusted for gender and age. Models with reduction of symptoms (50% or over reduction in PHQ-9) as dependent variable adjusted for gender, comorbidities and presence of major depression. Models with quality adjusted life years (QALY) as dependent variable adjusted for gender, age and baseline quality of life.

#### Societal perspective

**Figure 2:**
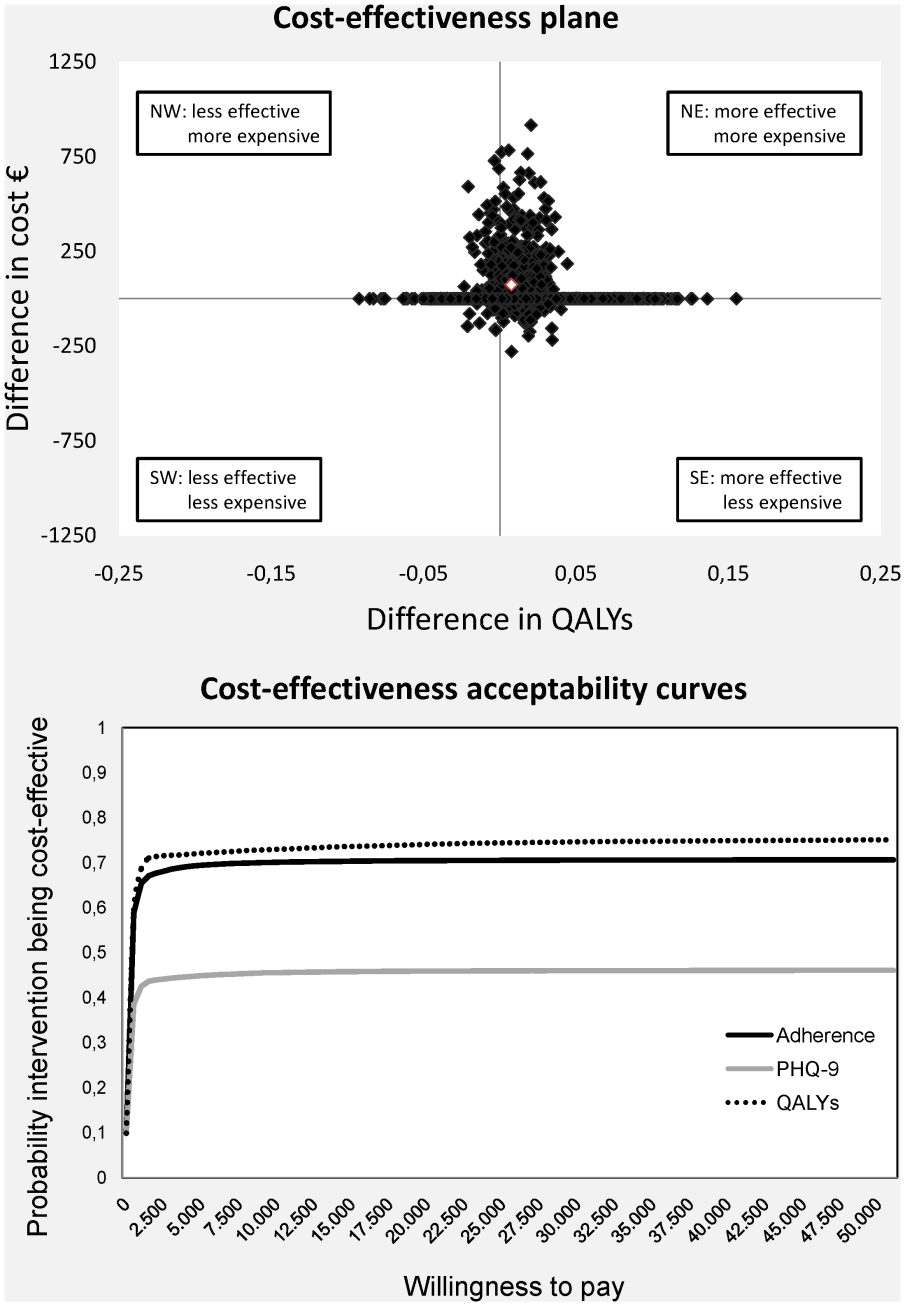
Cost-effectiveness plane for adherence with antidepressant therapy (pharmacist intervention vs usual care) and cost-effectiveness acceptability curves for adherence with antidepressant therapy, remission of depressive symptoms (PHQ-9) and QALYs estimated using bootstrapping from the societal perspective. The central white dot in the cost-effectiveness plane indicates the point estimate of the incremental cost-effectiveness ratio.

The bootstrapped cost-effectiveness pairs for the CPI effects on adherence were primarily located in the northeast (64%) and northwest (26%) quadrant, indicating that the costs in the CPI group were higher but that adherence did not differ between groups (Figure 2). The ICER indicated that €1,866 needs to be invested per extra adherent patient (Table 4).

Similar results were found for QALYs (Table 4). The CPI group showed both higher costs and a small increase in terms of QALYs compared with UC, resulting in an ICUR of €9,872.

Whereas costs were higher, the CPI group showed a negative improvement in the remission of depressive symptoms, resulting in a negative ICER (Table 4) (UC dominated the CPI).

The CEACs showed that the probability of the intervention being cost-effective was 0.71 if the society is willing to pay €17,000 for one extra adherent patient (Figure 2). In terms of remission of symptoms and QALY, if we take into account a WTP of €500 per extra remitted patient or QALY, the probability of the CPI being cost-effective in comparison with UC was 0.44 and 0.71, respectively.

If willingness to pay (WTP) is €30,000 per one extra adherent patient, per extra remission of symptoms or per QALY, the probability of the CPI being cost-effective was 0.71, 0.46 and 0.75, respectively.

#### Health system perspective

Since indirect costs were responsible for most of the difference in total societal costs between the groups, when the healthcare perspective was used the cost difference became smaller. As a result, the cost-effect pairs were more evenly distributed among the northern and southern quadrants in the CE plane (Table 4). The ICER was €962 per extra adherent patient for the CPI compared with UC.

The ICER was also smaller in terms of QALYs (€3,592 per extra QALY). In terms of remission of symptoms, the UC still dominated the CPI (€-3,946 per one extra remission).

From a health system perspective, the probability of the intervention being cost-effective is 0.71 if the WTP is €6,000 for an extra adherent patient and €100 for an extra QALY. If WTP is €30,000 per one extra adherent patient or QALY, the probability of the CPI being cost-effective was 0.71 and 0.76, respectively.

In terms of remission of symptoms, the maximum probability of CPI being cost-effective in comparison with UC (i.e. even if WTPis an infinite amount of money) was 0.46.

### Sensitivity analyses

Results of the sensitivity analyses were mainly in concordance with the main analyses and led to the same conclusions as the main analyses (Table 4).

In the PP analysis, costs differences were slightly larger than in the main analysis but effectiveness of the CPI was also larger in terms of adherence thus reducing the ICER (€1,455) and increasing the probability of CPI being cost-effective to 0.77 if WTP is €17,000. On the other hand, no difference in QALYs was observed in the PP analysis and consequently the ICER increased (€25,522).

The complete case and the DSM-IV criteria analysis showed smaller differences in adherence but the difference in costs was also reduced, not altering the results from the main analyses much.

Results of the sensitivity analyses in which the intervention costs were doubled or the average salary was used instead of the minimum salary did not differ from the main analysis.

## Discussion

### Main findings

The aim of the present study was to assess the cost-effectiveness after 6 months of a brief CPI compared to UC on the improvement of adherence, QALYs and clinical symptoms in primary care patients starting pharmacological treatment for depression.

The effectiveness analysis showed no statistically significant differences between groups in either adherence, depressive symptoms or QALYs. Total costs were higher in the CPI group, mainly as a consequence of increased costs in productivity losses. Cost-effectiveness planes and CEACs showed that a brief CPI had a probability of only 0.71 and 0.75 in terms of improvement of adherence and QALY, respectively, of being cost-effective when compared to UC. The CPI was unlikely to be cost-effective in comparison with UC in terms of remission of symptoms.

### Comparison with previous findings

Until now, to the best of our knowledge, only one study has been published on the cost-effectiveness of a pharmacist intervention for depression [17]. Bosmans et al conducted a randomized controlled trial in The Netherlands in which a pharmacist intervention plus a take-home educational videotape were compared to UC for patients with depression initiating treatment with antidepressants. Bosmans and colleagues found no impact of the intervention on the improvement of adherence or clinical symptoms of depression. In their study, total costs were slightly higher in the intervention group but the difference was not statistically significant. As was the case in the present study, in the study by Bosmans et al indirect costs accounted for most of the difference in total costs between groups, although the difference between groups in indirect costs was not statistically significant.

Bosmans et al found little evidence supporting the cost-effectiveneness of a brief educative pharmacist intervention into clinical practice, which is consistent with the results observed in the present paper.

Schoenbaum and colleagues evaluated the impact of support medication adherence program that was implemented via the telephone by trained practice nurses [41]. In this study, the intervention group generated higher costs but differences were not statistically significant. No statistically significant differences in QALYs were observed between the intervention and UC groups and ICER was €37,422 (adjusted to 2009 Euros). This result is in line with the results presented in the present paper showing that in terms of QALYs, low intensity educative interventions implemented by community pharmacists are not cost-effective in comparison with UC when dealing with depressed patients who start antidepressant treatment.

### Strengths and limitations

Economic evaluations are highly affected by sampling uncertainty. Size calculation was based on the improvement in adherence to antidepressants and the study could have been underpowered to detect differences in the cost-effectiveness analysis. However, to the best of our knowledge, our study has the largest sample size used to evaluate the cost-effectiveness of a pharmacist intervention in depressed patients.

Second, patients in the intervention and control group attended the same community pharmacies and contamination of the control group could have occurred. This could have been prevented by performing a cluster randomization at the pharmacy level. To minimise the impact of this contamination, pharmacists were asked to be aware of contamination when attending patients in the UC instead than at the patient level group and patients were asked not to share information with other patients participating in the study.

Third, the GPs that participated in the study could have had a special interest in the topic under study and could have routinely conducted interventions to improve patients' adherence to antidepressants. Although this would have affected both UC and CPI groups, it could have limited the margin of improvement of the pharmacist intervention.

Fourth, a follow-up period of 6 months may be too short to be able to evaluate long term costs and effects of the intervention such as relapse of depression. Higher rates of adherence have been associated to a lower risk of relapse which could reduce the costs [12].

An important strength is that this was a naturalistic study with very wide inclusion criterion, which was conducted in two different populations and where the intervention was implemented by many different community pharmacists. This increases the generalisability of the results while this also could have introduced heterogeneity. The proportion of men and women differed between groups and only half of the sample met DSM-IV criteria for major depression. However, with exception of the unadjusted analysis, all the models were controlled for gender and the presence of major depression was tested as an adjusting variable in all the models and included when necessary. Also, a sensitivity analysis of the patients that presented major depression according to DSM-IV criteria has been included. Moreover, patients could decide whether to refill their prescription and move from one to another pharmacy in successive visits. Consequently, 26% of the patients in the intervention group did not receive the intervention as allocated. However, we think that this is representative of daily clinical practice and that this greatly improves the generalisability of the results.

Finally, the main clinical outcome, adherence to antidepressants, was measured using electronic pharmacy records. Patients could have refilled prescription but not take them. However, this method has two advantages: patients are unaware of the fact that their adherence to medication is being observed and information can be collected even when patients drop-out from the study, avoiding missing data for our primary outcome.

## Conclusions

A brief CPI to improve adherence to antidepressants in patients initiating pharmacological treatment for depression, after 6 months follow-up, showed a maximum probability of being cost-effective of 0.71 and 0.75 in terms of improvement of adherence and QALY, respectively, when compared to UC. In view of the available evidence, we cannot recommend regular implementation of low intensity pharmacist interventions addressed to improve adherence to antidepressants in depressed patients.

The cost-effectiveness of more complex pharmacist's interventions needs to be evaluated before its implementation. In future studies, a longer follow-up period and the use of cluster randomization that limits contamination is recommended. Considering the uncertainty surrounding the costs in the sample size calculations is also necessary.

## Supporting Information

Checklist S1 **CONSORT Checklist.**
(DOC)

Protocol S1 **Trial Protocol.**
(PDF)
