# Correction: Cost-Effectiveness of a Community Pharmacist Intervention in Patients with Depression: A Randomized Controlled Trial (PRODEFAR Study)

**DOI:** 10.1371/journal.pone.0147459

**Published:** 2016-01-14

**Authors:** Maria Rubio-Valera, Judith Bosmans, Ana Fernández, Maite Peñarrubia-María, Marian March, Pere Travé, Juan A. Bellón, Antoni Serrano-Blanco

The following information is missing from the Funding section: Red de Investigacion en Actividades Preventívas y Promoción de la Salud (Research Network on Preventative Activities and Health Promotion) (Instituto de Salud Carlos III, RD06/0018/0017) and the European Regional Development Fund.

## References

[1] Rubio-Valera M, Bosmans J, Fernández A, Peñarrubia-María M, March M, Travé P, et al (2013) Cost-Effectiveness of a Community Pharmacist Intervention in Patients with Depression: A Randomized Controlled Trial (PRODEFAR Study). PLoS ONE 8(8): e70588 doi: 10.1371/journal.pone.0070588 23950967 10.1371/journal.pone.0070588 PMC3741197

